# Effects of Microecological Preparations on Obese Patients after Bariatric Surgery: A Systematic Review and Meta-Analysis

**DOI:** 10.1155/2020/8724546

**Published:** 2020-05-31

**Authors:** Hanfei Zhu, Ziqi Ren, Yan Zang, Hongxia Hua, Jinling Lu, Qin Xu, Shuqin Zhu

**Affiliations:** ^1^School of Nursing, Nanjing Medical University, Nanjing 210000, China; ^2^Department of Bariatirc and Metabolic Surgery, The First Affiliated Hospital of Nanjing Medical University, Nanjing 210000, China

## Abstract

Vitamin deficiency, bacterial overgrowth, and gastrointestinal symptoms can be detected in obese patients after bariatric surgery that influences their quality of life (QoL) and weight. It is unclear if microecological preparations benefit obese patients following bariatric surgery. The aim of this study is to investigate the effects of microecological preparations on QoL, excess weight loss (EWL), and levels of vitamin B_12_ and inflammatory markers. We searched seven databases to identify reports published till December 1, 2019, and included randomized controlled trials investigating the effects of microecological preparations in obese adults undergoing bariatric surgery. The primary outcomes included QoL and EWL, while secondary outcomes comprised serum levels of vitamin B_12_, interleukin 6, TNF-*α*, and C-reactive protein (CRP). Study bias was analyzed using the Cochrane risk-of-bias tool. Statistical analyses were performed using Review Manager. The mean difference in outcomes was calculated using standardized mean difference (SMD) with a confidence interval (CI) of 95%. A majority of the studies showed a low or moderate risk of bias. Meta-analysis showed significantly higher levels of vitamin B_12_ in postoperative patients administered with microecological preparations (SMD = 0.52; 95% CI = 0.08–0.95; *P* = 0.02). There were no significant differences in QoL (SMD = −0.14; 95% CI = −0.45–0.17; *P* = 0.38), EWL (SMD = 0.45; 95% CI = −0.16–1.05; *P* = 0.15), and levels of TNF-*α* (SMD = −0.29; 95% CI = −0.64–0.05; *P* = 0.09), interleukin 6 (SMD = −0.1; 95% CI = −0.81–0.61; *P* = 0.78]), and CRP (SMD = 0.02; 95% CI = −0.32–0.36; *P* = 0.93). The trials examined indicated that microecological preparations had limited efficacy in improving QoL, EWL, and inflammatory response, but they stimulated the synthesis of vitamin B_12_. This may help in designing efficient microecological preparations to supplement bariatric surgery in obese individuals.

## 1. Introduction

Obesity is a global health concern associated with physical and psychological conditions that present challenges for the healthcare industry. The World Health Organization has reported that more than 39% and 13% of adults were overweight and obese, respectively, in 2016 [1]. Individuals with a body mass index (BMI) of ≥35 kg/m^2^ are considered morbidly obese. Obesity leads to individuals developing hypertension [2], diabetes mellitus [3], cardiovascular disease [4], and kidney disease [5]. Moreover, obesity is a socioeconomic burden that is associated with increased medical costs for the treatment of related diseases [6]. Thus, the timely and efficient treatment of obesity is imperative. Bariatric surgery is currently used as the most effective and reliable method to treat morbid obesity and comorbidities [7]. Sleeve gastrectomy (SG) and Roux-en-Y gastric bypass (RYGB) are commonly used techniques in bariatric surgery that include implementing limited food intake, enhancing the passage of chymus into the distal small intestine, and altering the release of gastrointestinal hormones [8]. Gut microbiota play an important role in regulating host glucose metabolism. Bariatric surgery reduces the abundance of gut microbiota and triggers changes in microbial composition. An increase and decrease in the abundance *Bacteroidetes* and *Firmicutes*, respectively, are associated with weight loss [9]. However, patients who have undergone bariatric surgery are associated with modifications in the composition of gut microbiota that result in vitamin deficiency, bacterial overgrowth, and gastrointestinal disorders, thereby influencing postoperative clinical outcomes [10].

Microecological preparations, such as probiotics, prebiotics, and synbiotics, comprise one of the safe approaches to improve the composition of gut microbiota in humans [11] that positively regulate inflammatory bowel disease [12], infection by *Clostridium difficile* [13], immune responses [14], and diabetes [15]. Probiotics reduce body weight, BMI, and fat percentage in overweight or obese individuals [16]. The International Scientific Association classifies probiotics as active microorganisms prepared from a single or multiple strains of bacteria with low or no pathogenicity that benefit host health of the host organism [17]. The preparations primarily comprise *Bifidobacterium*, *Lactobacillus*, and Gram-positive cocci, such as *Streptococcus faecalis*. In contrast, prebiotics are substances that can be selectively utilized by host microbiota to benefit host health [17]. Synbiotics are synergistic combinations of prebiotics and probiotics [18]. Patients administered microecological preparations following bariatric surgery show benefits in their quality of life (QoL), excess weight loss (EWL), vitamin accessibility, and inflammatory marker expression. Karbaschian et al. [19] showed that probiotics stimulate weight loss in patients undergoing gastric bypass. However, another study [20] showed that administering probiotics does not improve anthropometric measurements in patients following laparoscopic SG. Furthermore, the effects of probiotics on the QoL, vitamin availability, and inflammation remain unclear [19–22]. An integrative review [23] described the impact of probiotics to reduce the gastrointestinal symptoms in postoperative patients but lacked the use of systematic retrieval methods and quality assessment of the literature. Thus, owing to this gap in knowledge, we systematically evaluated the efficacy of microecological preparations on clinical outcomes in adults following bariatric surgery.

## 2. Materials and Methods

### 2.1. Data Search

Two independent researchers utilized PubMed (Medline), Embase, Web of Science, Cochrane Controlled Register of Trials (CENTRAL), Proquest, Scopus, and Cumulative Index of Nursing and Allied Health Literature (CINAHL Complete) according to PRISMA (Preferred Reporting Items for Systematic Reviews and Meta-Analyses) guidelines [24]. The last date of research included was December 1, 2019. Search terms included Mesh headings and keywords related to bariatric surgery and microecological preparations (Supplementary Materials). The search was not refined using filters. We also searched published studies and the relevant gray literature manually to avoid omissions.

### 2.2. Inclusion Criteria

We selected random clinical trials (RCTs) for meta-analysis. Quasi-RCTs and non-RCTs were excluded. We included obese adults (age ≥18 years) who had undergone bariatric surgery (a type of surgery was not restricted). The experimental group was treated with microecological preparations (probiotics, prebiotics, synbiotics, or a combination of two preparations). The control group was subjected to placebo or conventional treatment. The primary outcomes comprised QoL and EWL. The secondary outcomes included vitamin B_12_ and inflammatory markers, such as C-reactive protein (CRP), interleukin 6 (IL-6), and tumor necrosis factor-*α* (TNF-*α*). Trials with at least one outcome of interest were included. Exclusion criteria included duplicate publications or unavailable full text from the included trials and article language except English or Chinese.

### 2.3. Data Extraction

Data were extracted independently, including the first author, country, published year, sample size, age, gender, type of surgery, type of microecological preparations administered, daily dose, and patient outcomes. For eligible articles with unclear information, the corresponding author was e-mailed to request additional explanations. Research with unavailable data was not included in the meta-analysis [25]. Missing standardized differences were derived from other statistical methods and data from each subgroup were merged according to the Cochrane handbook [26]. Since most of the data were available in the short term, we investigated the main short-term effect of microecological preparations.

### 2.4. Risk of Bias Assessment

To evaluate the methodological quality of the studies included, the risk of bias was assessed according to the Cochrane Handbook for Systematic Reviews of Interventions V5.1.0 [26]. We analyzed the risk of occurrence of the six domains of bias: selection, performance, detection, attrition, reporting, and other types of bias. Two independent investigators classified the six studies into high, low, or unclear risk of bias. Disagreements between investigators were resolved by discussion or by a third investigator until they reached a consensus.

### 2.5. Statistical Analyses

All of the statistical analyses were performed using Review Manager (RevMan V.5.3; Cochrane Collaboration, Oxford, UK). A two-tailed *P* value of <0.05 was considered statistically significant. The mean difference in outcomes between the intervention and control groups of each study was calculated using standardized mean difference (SMD) with a confidence interval (CI) of 95% owing to the multiple units of measurement [27]. SMD was estimated using a fix-effect model for studies with no heterogeneity; the random effect model was employed for the other studies. Heterogeneity was assessed by the Cochrane *Q*-test and *I*^*2*^ statistic (degree of heterogeneity). Moderate-to-high heterogeneity was categorized based on the *P* value from the *Q*-test (<0.1) and/or *I*^*2*^ (<50%) [27]. Subgroup and sensitivity analyses using study- and patient-level characteristics were performed to explore the origin of the heterogeneity. Sensitivity analyses were also performed by omitting one trial at a time from the included studies to assess its effect on the SMD. Funnel plots were used to investigate publication bias if sufficient studies (>10) were included [26].

## 3. Results

### 3.1. Database Search

**Figure 1:**
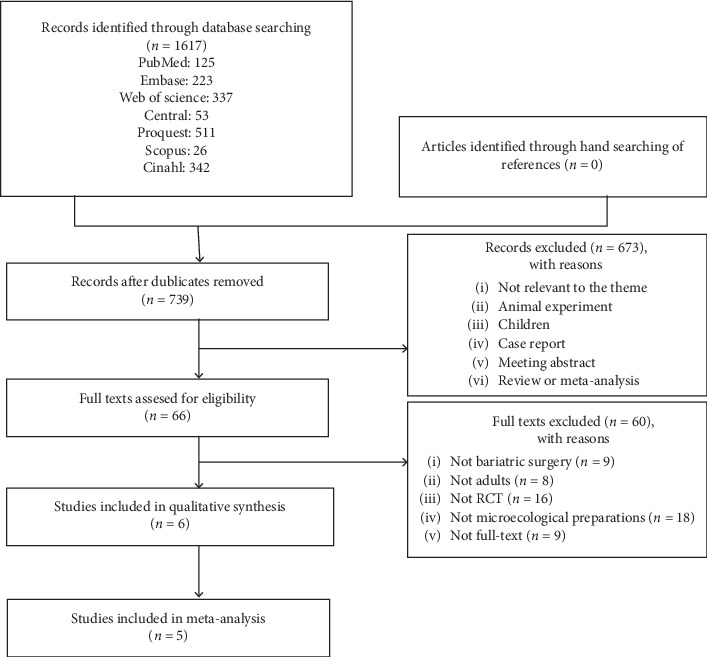
Identification and selection of the relevant literature.

We identified 1,617 articles, of which 878 duplicate and 673 irrelevant articles (based on titles and abstracts) were excluded. We then screened the full-text and excluded 60 articles for the following reasons: the subjects did not undergo bariatric surgery (*n* = 9), animals (*n* = 5), or children (*n* = 3); it was a non-RCT study (*n* = 16); the study did not use microecological preparations (*n* = 18); full-text article was not available (*n* = 9). Finally, 6 articles were included [19–22, 25, 28].  Figure 1 shows the flow chart for the selection process.

### 3.2. Study Characteristics

The six trials included were published from 2008 to 2019 and performed in the United States of America, Brazil, China, Iran, and Israel. All the study individuals were morbidly obese (BMI ≥35 kg/m^2^) who underwent RYGB, vertical SG, and one-anastomosis gastric bypass. The studies reported data on 269 patients. The number of patients within each study ranged from 9 to 80 years, and follow-up period varied between 2 weeks and 13 months. Among the six studies, 4, 1, and 1 used probiotics, synbiotics, and a combination of prebiotics and synbiotics, respectively.  Table 1 summarizes the main characteristics of the study.

### 3.3. Assessment of Risk of Bias

**Figure 2:**
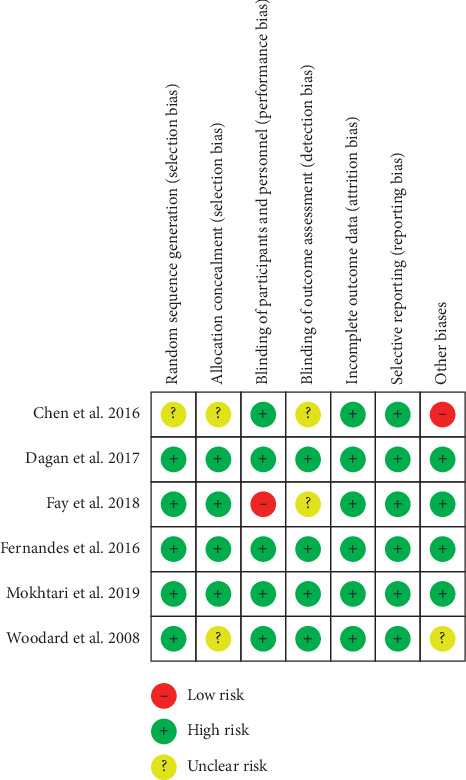
Risk of bias.

**Figure 3:**
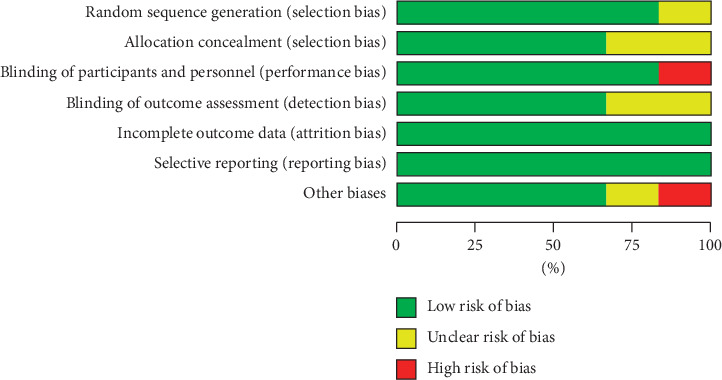
Proportion of risk of bias.

Figures 2 and 3 show the quality assessment of included studies using Cochrane Collaboration. All the articles were assessed with low risks in random sequence generation except one trial [25]. However, this article did not provide information about an appropriate randomization procedure and allocation concealment. Similarly, two other trials did not describe the allocation concealment strategy [25, 28]. Therefore, they were classified under “unclear risk” based on selection bias. In all the trials, attrition and reporting bias were unidentified. Only one report [22] performed a single-blind trial on patients without blinding the researchers and study personnel; thus, it was considered as “high risk” in performance bias. Two trials conducted by Kazzi [22] and Chen [25] did not specify whether the outcome measurer was blinded and were categorized with an “unclear risk” in detection bias. One trial described patient age, sex, and BMI and lacked baseline comparability [25]. Another such trial did not include sufficient information to determine if it was free of other biases [28]. The quality of three studies [19–21] was grade A, and probability of bias was low. The remaining three studies [22, 25, 28] were grade B with a moderate probability of bias.

### 3.4. Primary Outcomes

#### 3.4.1. Quality of Life

**Figure 4:**
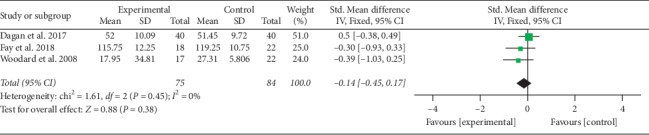
Forest plot for the quality of life.

Three trials were included in the meta-analysis to estimate the effect of microecological preparations on QoL. Microecological preparations did not improve patient QoL after bariatric surgery (SMD = −0.14; 95% CI = −0.45–0.17; *P*=0.38) (Figure 4). No significant heterogeneity was detected among the three studies (Χ^*2*^ = 1.61;*P*=0.45; *I*^*2*^ = 0%).

#### 3.4.2. Excess Weight Loss

**Figure 5:**
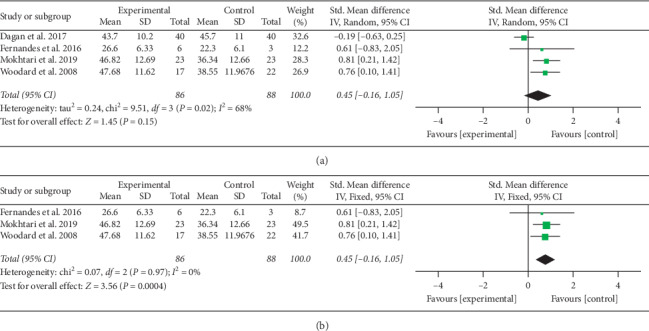
(a) Forest plot for excess weight loss (%).(b) Forest plot for excess weight loss (%) using subgroup analysis of surgery types.

Meta-analysis based on four RCTs suggested that microecological preparations did not result in an increase in EWL% in patients who underwent bariatric surgery (SMD = 0.45; 95% CI = −0.16–1.05; *P*=0.15) (Figure 5(a)). Moderate heterogeneity was observed across the four trials (Χ^*2*^ = 9.51; *P*=0.02; *I*^*2*^ = 68%). Specifically, subgroup analysis of the surgery types showed that microecological preparations reduced more weight in patients who underwent gastric bypass surgery (SMD = 0.77; 95% CI = 0.35–1.20; *P*=0.0004) (Figure 5(b)).

#### 3.4.3. Vitamin B_12_

**Figure 6:**
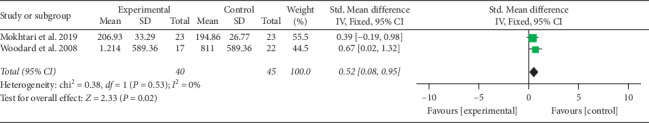
Forest plot for serum levels of vitamin B_12_.

Two trials comprising 40 participants were included in the meta-analysis to determine the effect of microecological preparations on the serum levels of vitamin B_12_. We observed that the microecological preparations increased serum levels of vitamin B_12_ compared to that in the placebo group (SMD = 0.52; 95% CI = 0.08–0.95; *P*=0.02) (Figure 6). No significant heterogeneity was found between the two studies (Χ^*2*^ = 0.38; *P*=0.53; *I*^*2*^ = 0%).

#### 3.4.4. Inflammatory Markers

**Figure 7:**
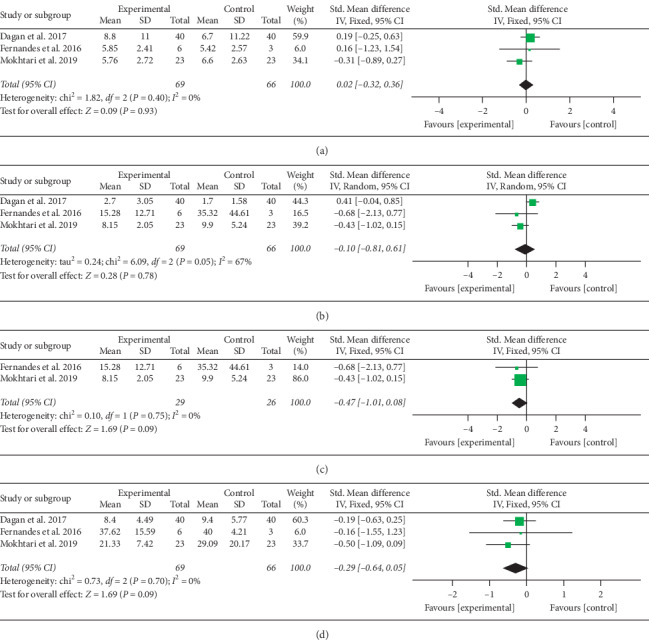
(a) Forest plot for C-reactive protein. (b) Forest plot for the levels of interleukin 6. (c) Forest plot for interleukin-6 levels after subgroup analysis of surgery types. (d) Forest plot for tumor necrosis factor-*α*.

The meta-analysis of three trials consisting of 69 participants was not significant for the levels of inflammatory markers (CRP, IL-6, and TNF-*α*; Figures 7(a)–7(d). Moderate heterogeneity was found across the three trials (Χ^*2*^ = 6.09; *P*=0.05; *I*^*2*^ = 67%). Excluding Dagan et al. resulted in a loss of heterogeneity (*I*^*2*^ = 0%; *P* = 0.09) and significance (SMD = −0.47; 95% CI = −1.01–0.08; *P*=0.09) of IL-6 levels between the studies (Figure 7(c)).

#### 3.4.5. Sensitivity Analyses and Publication Bias

Excluding the studies one by one did not significantly alter the pooled effects of microecological preparations on QoL, EWL, and levels of vitamin B_12_ and inflammatory markers, indicating that the results were consistent after adjustments. Owing to the insufficient number of studies included (<10), we could not analyze publication bias by a funnel plot. This is because when there are fewer studies, the power of the tests is too low to distinguish chance from real asymmetry.

## 4. Discussion

In this study, we analyzed the correlation between the effects of microecological preparations and bariatric surgery in human adults. To the best of our knowledge, this is the first meta-analysis to provide comprehensive insight on the effects of microecological preparations for obese patients having undergone bariatric surgery.

We observed that probiotics, prebiotics, or synbiotics could not improve the QoL in postoperative patients. This could be attributed to the enhancement in short- and long-term QoL by surgery [29]. However, microecological preparations have been reported to improve dyspepsia and reduce gastrointestinal symptoms in postoperative patients [25]. A study reported that abdominal symptoms after bariatric surgery were common and required further research [30]. Microecological agents may alleviate these symptoms by improving the function of the intestinal barrier and maintaining the integrity of intestinal epithelial cells [31]. Therefore, the effect of microecological agents on gastrointestinal symptoms requires detailed research in the future.

The effect of microecological agents on weight loss has been shown in animal models. Tremaroli demonstrated that transplanting gut microbiome from patients after RYGB reduced the deposition of fat in germ-free mice [32]. Sprague-Dawley rats that have undergone duodenal-jejunal bypass surgery showed an increase in weight loss after being administered probiotics [33]. However, a meta-analysis reported that oral supplementation of probiotics or synbiotics could not reduce body weight or BMI in overweight and obese adults [31]. Similarly, we did not observe an effect of microecological preparations on EWL in this meta-analysis. Subgroup analysis showed that it may be useful in the short term in patients having undergone RYGB or OAGB, but no long-term benefits have been found in RCTs till date. Compared with SG, the composition of gut microbiota was dramatically altered in patients having undergone gastric bypass [34]. We speculate that microecological preparations improved the gut microbiota ecosystem and increased weight loss.

Although these trials were classified as having a low or moderate risk of bias, the benefits of microecological preparations on inflammatory markers were not observed in this meta-analysis. This contradicts the reported effects of synbiotics in immunomodulation that improve metabolic endotoxemia or low-grade inflammation in obese people [35]. Endotoxin concentrations reduced along with hs-CRP or CRP concentrations, suggesting that synbiotics modulate the inflammatory response and promote metabolic derangements. This is mediated by gut microbiota by promoting antimicrobial activity and enhancing barrier function or immunomodulation. Probiotics that specifically impact body weight and metabolism are under investigation. However, *Lactobacillus* and *Bifidobacterium* have antiobesity properties based on a recent experiment-based study [36]. A review showed that the abundance of *Escherichia* and *Akkermansia* increases with a decrease in the content of *Bifidobacterium, Blautia, Dorea,* and *Lactobacillus* after bariatric surgery, thereby affecting insulin sensitivity and decreasing inflammation [37]. Thus, the diversity in the phylum of bacteria present, namely, the ratio of *Firmicutes/Bacteroidetes*, changes; *Firmicutes* and *Bacteroidetes* share a symbiotic relationship that affects energy intake, transformation, and storage [38]. The anti-inflammatory effects of probiotics or prebiotics in postoperative patients cannot be neglected since it alters the composition of gut microbiota. However, how the microbiota can be modified remains unknown since most of the studies could not detect microbial content except Dagan et al. [20].

Our meta-analysis showed improved serum levels of vitamin B_12_ upon the administration of microecological preparations that can be attributed to the synthesis of vitamin B_12_ by lactic acid bacteria [39]. Several studies have shown vitamin B_12_ deficiency in patients who underwent bariatric surgery [40]. The American Society for Metabolic and Bariatric Surgery updated the guidelines indicating the importance of supplementing micronutrients in patients after bariatric surgery [41]. Taken together, probiotic supplements may be a new approach in promoting serums levels of vitamin B_12_. However, the mechanism involved remains unclear.

To the best of our knowledge, this is the first study to systematically review and analyze the effects of microecological preparations in patients who have undergone bariatric surgery. The comprehensive literature search involving 7 electronic databases and manual search for references provided an advantage to this study over similar reports. Moreover, the studies included in our meta-analysis were associated with a low or moderate risk of bias with good quality of data. However, this meta-analysis has several limitations. First, the small sample size and number of included studies limited statistical analysis. Second, the study by Dagan et al. [20] was found to be the main source of heterogeneity because of the surgery type used. Dagan et al. [20] reported that the administration of probiotics did not improve inflammatory responses and weight loss in patients having undergone SG. Consequently, upon the exclusion of this study from the meta-analyses, microecological preparations augmented weight loss after gastric bypass. Thus, we could not determine the role of microecological preparations based on the type, dose, and duration of treatment. Third, the results of our meta-analysis are relevant only for short-term outcomes, leading to the loss of statistical significance in some results.

## 5. Conclusions

Based on the available reports, this meta-analysis has demonstrated that microecological preparations improved the short-term serum levels of vitamin B_12_ but did not affect the QoL, EWL, and inflammatory markers (CRP, IL-6, and TNF-*α*). Future research should be designed to investigate the detailed effects of microecological preparations in postoperative patients and account for dietary intake, physical activity, controlled lifestyle factors, setting strict standards for participants (excluding those having been administered antibiotics or drugs that may skew the results), and employing a sample size for sufficient statistical significance. Efficient detection of microbiota is imperative to further explore the correlation between the effects of microecological agents and bariatric surgery mediated by gut microbiota. It is also important to evaluate the altered profiles of gut microbiota after supplementation with various microecological preparations. Identifying the specific strains involved with conferring host benefit is also crucial. Finally, the dosage, duration of treatment, and long-term effects of the administering various microbial strains need to be investigated. This will help in devising efficacious combinations or lone preparations of probiotics, prebiotics, and/or synbiotics in positively regulating postoperative patient health.

## Figures and Tables

**Table 1:** Characteristics of included trials.

Study (year, country)	Total patients, (F : M; age, mean)	Type of surgery	Supplement	Study component	Dose, treatment duration	Results
Woodard et al. [28] (2008, USA)	41, I: (20/2; 41.2) C:(16/3; 48.6)	LRYGB	Probiotics	*Lactobacillus* (2.4 × 10^8^ CFU)	Once a day, 6 months	⟷QoL ↑EWL%^*∗*^ ⟷hsCRP ↑Vitamin B_12_^*∗*^
Mokhtari and Slizewska [19] (2019, Iran)	46, I: (23; 32.35) C: (23; 36.95)	OAGB	Synbiotics	*Lactobacillus casei* (3.5 × 10^9^ CFU/g), *Lactobacillus rhamnosus* (7.5 × 10^8^ CFU/g), *Streptococcus thermophilus* (1 × 10^8^ CFU/g), *Bifidobacterium breve* (1 × 10^10^ CFU/g), *Lactobacillus acidophilus* (1 × 10^9^ CFU/g), *Bifidobacterium longum* (3.5 × 10^9^ CFU/g), *Lactobacillus bulgaricus* (1 × 10^8^ CFU/g), and FOS 38.5 mg	Once a day, 4 months	↑EWL%^*∗*^ ↓TNF-*α*^*∗*^ ⟷ IL-6, hs-CRP ⟷Vitamin B_12_ ↑25(OH)*D*_3_
Fernandes et al. [21] (2016, Brazil)	9, prebiotic group: (2/1; 36.7) synbiotic group: (3; 42.0) C:(3; 32.0)	RYGB	Prebiotics and synbiotics	Prebiotic: FOS 6 g Synbiotics: FOS and *Lactobacillus paracasei*(1 × 10^9^ CFU), *Lactobacillus rhamnosus* (1 × 10^9^ CFU), *Lactobacillus acidophilus* (1 × 10^9^ CFU), and *Bifidobacterium lactis* (1 × 10^9^ CFU), 6 g	Once a day, 15 days	⟷EWL% ⟷ IL-6; TNF-*α*; CRP
Dagan et al. [20] (2018, Israel)	80, I: (24/16; 42.1) C:(22/18; 44.2)	LSG	Probiotics	11 different species of bacteria (>5 × 10^9^ CFU)	Twice a day, 6 months	⟷QoL ⟷ EWL% ⟷CRP; TNF-*α*; IL-6; IL-10 ↓GI symptoms: Constipation
Fay et al. [22] (2018, USA)	40, I:(12/6; 52.7) C:(19/3; 44.1)	LSG	Synbiotics	*Bacillus coagulans (*4.5 × 10^8^ CFU) and galactomannans (300 mg)	Once a day, 3 months	⟷QoL
Chen et al. [25] (2016, China)	53, group A: (14/5, 36.1) group B: (13/5, 34.8) C: (10/6, 34.4)	RYGB, MGB, and SG	Probiotics	Group A *Clostridium butyricum* (5 × 10^8^ CFU); Group B *Bifidobacterium longum* (8 × 10^8^ CFU)	Twice a day, 2 weeks	⟷QoL ↓GI symptoms Abdominal pain, bloating, and noises; excessive passage of gas; foul smelling flatulence; belching; heartburn

LRYGB, laparoscopic Roux-en-Y gastric bypass; RYGB, Roux-en-Y gastric bypass; LSG, laparoscopic sleeve gastrectomy; SG, sleeve gastrectomy; OAGB, one-anastomosis gastric bypass; MGB, minigastric bypass; I, intervention group; C, control group; CFU, colony-forming units; FOS, fructo-oligosaccharide; GI symptoms, gastrointestinal symptoms; QoL, quality of life; EWL%, percentage of excess weight loss; IL-6, interleukin 6; TNF-*α*, tumor necrosis factor-*α*; CRP, C-reactive protein. ⟷ indicates no significant difference between the intervention and control groups during the follow-up period; ↓ indicates significantly lower in the intervention group as compared to that in the control group during the follow-up period; ↑ indicates significantly higher in the intervention group than that in the control group during the follow-up period. ^∗^The intervention group data differed significantly from data of the control group in the third month after surgery, but not significantly different during the follow-up period.

## Supplementary Materials

Supplementary Materials See full search strategy in the Supplementary Material for comprehensive search.
