# Supplementary material for: Effects of Microecological Preparations on Obese Patients after Bariatric Surgery: A Systematic Review and Meta-Analysis
Source: Evid Based Complement Alternat Med. 2020 May 31;2020:8724546. doi: 10.1155/2020/8724546 (PMC7281838; doi:10.1155/2020/8724546)
Supplement: Supplementary Materials — See full search strategy in the Supplementary Material for comprehensive search. [file 8724546.f1.docx]

| PubMed (Medline) |
| --- |
| Search hits 125  (Probiotics[MeSH Terms] OR Prebiotics[MeSH Terms] OR Synbiotics[MeSH Terms] OR Lactobacillus[MeSH Terms] OR Bifidobacterium[MeSH Terms] OR “Yeast, Dried” [MeSH Terms] OR probiotic*[tw] OR prebiotic*[tw] OR synbiotic*[tw] OR lactobacil*[tw] OR bifidobacter*[tw] OR “dried yeast”[tw] OR postbiotic*[tw]OR “microecological preparation”[tw] OR enterobacil*[tw] OR streptococcus[tw] OR prevotella[tw] OR propionate[tw] OR inulin[tw] OR fructo-oligose[tw] OR fructan*[tw] OR fructooligosaccharide*[tw] OR fructo- oligosaccharide*[tw] OR galactooligosaccharide*[tw] OR galacto-oligosaccharide*[tw] OR oligofructose[tw] OR “short chain fatty acid” [tw] OR SCFA[tw] OR acetic[tw] OR butyric[tw] OR yogurt[tw])  AND  (bariatric surgery[MeSH Terms] OR Bariatric Surgery[Title/Abstract] OR gastric bypass[Title/Abstract] OR weight loss surgery[Title/Abstract] OR weight reduction surgery[Title/Abstract] OR metabolic surgery[Title/Abstract] OR obesity surgery[Title/Abstract] OR Roux-en-Y gastric bypass[Title/Abstract] OR sleeve gastrectomy[Title/Abstract] OR gastric sleeve[Title/Abstract] OR gastric banding[Title/Abstract] OR bariatric operation[Title/Abstract] OR duodenal-jejunal bypass[Title/Abstract] OR DJB[Title/Abstract] OR LAGB[Title/Abstract] OR LSG[Title/Abstract] OR RYGB[Title/Abstract]) |
| EMBASE |
| Search hits 223  ('probiotic agent'/exp OR 'probiotic agent' OR 'synbiotic agent'/exp OR 'synbiotic agent' OR 'prebiotic agent'/exp OR 'prebiotic agent' OR 'lactobacillus'/exp OR lactobacillus OR 'bifidobacterium'/exp OR bifidobacterium OR 'dried yeast'/exp OR 'dried yeast' OR 'fructose oligosaccharide' OR 'galactose oligosaccharide' OR 'inulin' OR ‘SCFA’ OR ‘acetic’ OR ‘butyric’ OR 'yoghurt') AND 'bariatric surgery' |
| Web of Science |
| Search hits 337  #1. TS = (probiotic* OR prebiotic* OR synbiotic* OR lactobacillus OR “dried yeast” OR Bifidobacterium OR postbiotic* OR “microecological preparation” OR enterobacillus OR streptococcus OR fructan* OR fructooligosaccharide* OR fructo- oligosaccharide* OR oligofructose OR galactooligosaccharide* OR galacto-oligosaccharide* OR prevotella OR propionate OR inulin OR fructo-oligose OR SCFA OR acetic OR butyric OR yogurt)  #2. TS = ("bariatric surgery" OR "obesity surgery*" OR "gastric bypass*" OR "weight loss surgery*" OR "weight reduction surgery*" OR “metabolic surgery” OR “obesity surgery” OR "Roux-en-Y gastric bypass*" OR "sleeve gastrectomy*" OR "gastric sleeve*" OR "gastric banding*" OR "bariatric operation*"OR "duodenal-jejunal bypass" OR LAGB OR DJB OR RYGB)  #3. #1 AND #2 |
| CENTRAL  Search hits 53 #1 probiotic* OR prebiotic* OR synbiotic* OR lactobacil* OR “dried yeast” OR bifidobacter* OR enterobacil* OR streptococcus OR fructan* OR fructooligosaccharide* OR fructo-oligosaccharide* OR oligofructose OR galactooligosaccharide* OR galacto-oligosaccharide* OR prevotella OR propionate OR inulin OR fructo-oligose OR SCFA OR acetic OR butyric OR yogurt:ti,ab,kw  #2 "bariatric surgery" OR "metabolic surgery" OR "bypass surgery""obesity surgery" OR "Gastric Bypass" OR "weight loss surgery" OR "weight reduction surgery" OR "Roux-en-Y Gastric Bypass" OR "sleeve gastrectomy" OR "gastric sleeve" OR "gastric banding" OR "bariatric operation" OR "duodenal-jejunal bypass" OR LAGB OR LSG OR RYGB:ti,ab,kw  #3 MeSH descriptor: [Probiotics] explode all trees #4 MeSH descriptor: [Prebiotics] explode all trees #5 MeSH descriptor: [Synbiotics] explode all trees #6 MeSH descriptor: [Postbiotics] explode all trees  #7 MeSH descriptor: [Lactobacillus] explode all trees #8 MeSH descriptor: [Bifidobacterium] explode all trees  #9 MeSH descriptor: [Yeast, Dried] explode all trees #10 #1 or #3 or #4 or #5 or #6 or #7 or #8 or #9 #11 MeSH descriptor: [Bariatric Surgery] explode all trees #12 #2 or #11 #13 #10 and #12 |
| ProQuest |
| Search hits 511  (probiotic* OR prebiotic* OR synbiotic* OR lactobacillus OR "dried yeast" OR Bifidobacterium OR postbiotic* OR "microecological preparation" OR enterobacillus OR streptococcus OR fructan* OR fructooligosaccharide* OR fructo- oligosaccharide* OR oligofructose OR galactooligosaccharide* OR galacto-oligosaccharide* OR prevotella OR propionate OR inulin OR fructo-oligose OR SCFA OR acetic OR butyric OR yogurt)  AND  ("bariatric surgery" OR "obesity surgery*" OR "gastric bypass*" OR "weight loss surgery*" OR "weight reduction surgery*" OR "metabolic surgery" OR "obesity surgery" OR "Roux-en-Y gastric bypass*" OR "sleeve gastrectomy*" OR "gastric sleeve*" OR "gastric banding*" OR "bariatric operation*" OR "duodenal-jejunal bypass" OR LAGB OR DJB OR RYGB) |
| SCOPUS |
| Search hits 26  (probiotic* OR prebiotic* OR synbiotic* OR postbiotic* OR lactobaci* OR bifidobacter*OR dried yeast OR “microecological preparation” OR enterobacil* OR streptococcus OR prevotella OR propionate OR inulin OR fructo-oligose OR fructan* OR fructooligosaccharide* OR fructo- oligosaccharide* OR oligofructose OR galactooligosaccharide* OR galacto-oligosaccharide* OR SCFA OR acetic OR butyric OR yogurt)  AND  ({bariatric surgery} OR {obesity surgery} OR {gastric bypass} OR {weight loss surgery} OR {weight reduction surgery} OR {metabolic surgery} OR {obesity surgery} OR {Roux-en-Y gastric bypass} OR {sleeve gastrectomy} OR {gastric sleeve} OR {gastric banding} OR {bariatric operation} OR {duodenal-jejunal bypass} OR LAGB OR DJB OR RYGB） |
| CINAHL Complete |
| Search hits 342  TX (probiotic* OR prebiotic* OR synbiotic* OR postbiotic* OR lactobaci* OR bifidobacter*OR dried yeast OR “microecological preparation” OR enterobacil* OR streptococcus OR prevotella OR propionate OR inulin OR fructo-oligose OR fructan* OR fructooligosaccharide* OR fructo- oligosaccharide* OR oligofructose OR galactooligosaccharide* OR galacto-oligosaccharide* OR SCFA OR acetic OR butyric OR yogurt)  AND  (“bariatric surgery” OR “obesity surgery” OR “gastric bypass” OR “weight loss surgery” OR “weight reduction surgery” OR “metabolic surgery” OR “obesity surgery” OR “Roux-en-Y gastric bypass” OR “sleeve gastrectomy” OR “gastric sleeve” OR “gastric banding” OR “bariatric operation” OR “duodenal-jejunal bypass” OR LAGB OR DJB OR RYGB） |
